# Towards Secure Internet of Things: A Coercion-Resistant Attribute-Based Encryption Scheme with Policy Revocation

**DOI:** 10.3390/e27010032

**Published:** 2025-01-02

**Authors:** Yuan Zhai, Tao Wang, Yanwei Zhou, Feng Zhu, Bo Yang

**Affiliations:** 1 School of Computer Science, Shaanxi Normal University, Xi’an 710062, China; zhaiyuan@snnu.edu.cn (Y.Z.); water@snnu.edu.cn (T.W.); zyw@snnu.edu.cn (Y.Z.); 2 State Key Laboratory of Integrated Services Networks, Xidian University, Xi’an 710119, China; 3 Koal Software Co., Ltd., 299, Jiangchang West Road, Jing’an District, Shanghai 200436, China; zhuf@koal.com

**Keywords:** internet of things, data privacy, deniable encryption, ciphertext-policy attribute-based encryption

## Abstract

With the development and application of the Internet of Things (IoT), the volume of data generated daily by IoT devices is growing exponentially. These IoT devices, such as smart wearable devices, produce data containing sensitive personal information. However, since IoT devices and users often operate in untrusted external environments, their encrypted data remain vulnerable to potential privacy leaks and security threats from malicious coercion. Additionally, access control and management of these data remain critical issues. To address these challenges, this paper proposes a novel coercion-resistant ciphertext-policy attribute-based encryption scheme. The scheme leverages chameleon hashing to enhance deniable encryption, achieving coercion resistance, thereby enabling IoT data to resist coercion attacks. Moreover, the scheme employs attribute-based encryption to secure IoT data, enabling fine-grained access control and dynamic user access management, providing a secure and flexible solution for vast IoT data. We construct the scheme on a composite order bilinear group and provide formal proofs for its coercion resistance, correctness, and security. Finally, through experimental comparisons, we demonstrate the efficiency and feasibility of the proposed scheme.

## 1. Introduction

The rapid development of IoT technology provides new models and tools for data collection, storage, processing, and analysis. For example, many people use smartwatches to monitor their heart rate, sleep quality, and daily activity levels. These devices can collect users’ physiological data in real time and store and analyze it through the cloud. The collection and use of similar personal privacy data have raised concerns about security and privacy protection in the IoT. In this context, data security and privacy protection become particularly important.

Attribute-based encryption (ABE) [1], as a cryptographic method that enables fine-grained access control, attracts widespread attention from IoT researchers [2]. Due to its ability to provide a more flexible and dynamic access management mechanism, ABE encryption technology is particularly well-suited for data sharing and access control in the IoT. For example, different devices and sensors may collect various types of data, and ABE technology can ensure that only users with the corresponding attributes can access a specific data set, thereby protecting the data from unauthorized access.

However, most existing ABE schemes generally assume that the random values and secret keys used in the encryption and decryption processes are kept confidential. This assumption implies vulnerability to coercion attacks, where an attacker may force the disclosure of these sensitive components, thereby undermining the security and practicality of the encryption scheme. When IoT device users exposed to untrusted external environments face coercion attacks, the encryption scheme used for IoT data may lose its effectiveness, thereby exposing users’ private data and severely compromising the secure use of IoT systems. Moreover, the exponential growth in the number of IoT devices has led to an explosive increase in IoT data, posing challenges for access management in ABE schemes. Specifically, the ability to dynamically revoke access authority from users whose qualifications have changed or lapsed is a critical functionality that must be addressed to ensure the ongoing security and relevance of ABE technology.

To address these challenges, this paper presents a novel ABE scheme that is specifically crafted to withstand coercion attacks. Our scheme is designed with the foresight that an adversary may force the disclosure of random numbers and secret keys. Nonetheless, it ensures semantic security even under such duress. This is accomplished by integrating a deniable encryption mechanism [3] that generates two distinct sets of secret keys: one for decrypting the actual ciphertext, and another, the fake key, for decrypting the ciphertext to a pre-determined false plaintext. In the event of a coercion attack, a user can disclose the fake key, which would decrypt the ciphertext to a fabricated message, thereby preserving the semantic security of the scheme under coercion. In addition, our proposed ABE scheme integrates an attribute revocation mechanism. This feature enables effective management of access rights, allowing IoT devices not only to withstand coercion attacks but also to promptly revoke the access attributes of users who no longer meet the criteria for data access. This dual capability enhances the security of IoT devices and the privacy of users’ personal data.

Specifically, the contributions of this paper are as follows.

- We propose a novel ciphertext-policy ABE (CP-ABE) scheme that combines chameleon hashing and deniable encryption techniques to achieve coercion resistance. In the face of coercion attacks, both IoT devices and users can provide false decryption results that convince the coercing party, thereby effectively protecting the real message and secret key and enhancing the coercion resistance of vulnerable IoT devices and users.
- The proposed scheme provides fine-grained access management for IoT data while incorporating flexible policy revocation functionality. This feature effectively protects sensitive data and enables dynamic management of user access rights, adapting to changing access requirements. We detail the specific construction methods based on composite order bilinear maps, leveraging advanced cryptographic primitives and mathematical tools to ensure the security and efficiency of the scheme while maintaining adaptability for large-scale deployments.
- We prove the correctness of the constructed scheme and conduct comprehensive security analysis and experimental comparisons. The analysis results indicate that our scheme excels in achieving coercion resistance and dynamic policy revocation functionalities. Experimental results demonstrate that, compared to existing schemes, our solution not only maintains a security advantage but also exhibits good performance, making it suitable for practical deployment and application in IoT systems.

### 1.1. Related Works

In this section, we introduce revocable CP-ABE and deniable encryption techniques. Furthermore, we discuss the applications of CP-ABE in the IoT and the issues it faces.

#### 1.1.1. Revocable CP-ABE

Hur and Noh [2], Liang et al. [4], and Xie et al. [5] proposed efficient CP-ABE schemes that enhance revocation capabilities through unique user identifiers, dual encryption methods, and outsourcing computation services. These approaches address the challenges of secure, fine-grained access control in data outsourcing systems. Building on this, Sahai et al. [6] introduced a novel concept of ciphertext delegation to address access control in cloud storage. Their approach allows a third party to re-encrypt ciphertexts to more restrictive policies without accessing secret keys, and it presents a fully secure revocable ABE scheme by modifying Lewko et al.’s approach [7]. This enables storage servers to update ciphertexts to revoke access for disqualified users while supporting dynamic key updates for selective user revocation.

Kim et al. [8] further advanced the field by converting CP-ABE into ciphertext-delegatable CP-ABE (CD-CP-ABE), which allows third parties to re-encrypt ciphertexts with stricter policies. Their approach features a generic delegation algorithm with linearly growing access structures, enhancing efficiency and applicability to existing CP-ABE schemes. Jiang et al. [9] addressed a key limitation in traditional ABE systems by proposing a scheme that supports efficient access policy updates without requiring re-encryption by the original encryptor. Their scheme allows for dynamic addition and revocation of attributes in access policies, using constant-size ciphertexts for decryption. Susilo et al. [10] introduced EACSIP, an extendable access control system with integrity protection for cloud environments. EACSIP enables secure extension of access policies to include new users without compromising data integrity, utilizing a novel cryptographic primitive called functional key encapsulation with equality testing.

Yu et al. [11] addressed attribute revocation in CP-ABE by integrating proxy re-encryption, enabling authorities to efficiently revoke user attributes through semi-trustworthy online proxy servers. Han et al. [12] proposed a CP-ABE scheme that enhances privacy and security with hidden access policies, revocation, and white-box traceability. Their scheme divides ciphertext into two parts: one for access policies (with only attribute names visible) and another for revocation information, managed by a binary tree, facilitating efficient user revocation and malicious user tracking. Ge et al. [13] introduced a revocable attribute-based encryption scheme with data integrity protection (RABE-DI) for cloud environments, ensuring that updated ciphertexts generated after revocation still correspond to the original plaintext, despite potential mistrust in cloud servers. Xue et al. [14] developed Poly-ABE, a CP-ABE scheme for multi-energy systems, offering fully hidden access policies, traceability, and revocability. By combining matrix-based access controls with a decryption permission binary tree, Poly-ABE improves privacy and security in integrated demand response models, efficiently managing data access and detecting malicious behavior.

#### 1.1.2. Deniable Encryption

Canetti et al. [3] introduced deniable encryption, enabling a sender to generate “fake” random choices to make a ciphertext appear as if it encrypts a different plaintext, thus preserving the real plaintext’s confidentiality even under coercion. They proposed schemes with polynomial deniability and demonstrated its applications in secure multiparty computation. Dürmuth and Freeman [15] advanced this concept by presenting the first sender-deniable public-key encryption scheme with a single encryption algorithm and negligible detection probability. Their construction allows the sender to convincingly fake an encrypted message without revealing the true plaintext, utilizing techniques based on quadratic residuosity and trapdoor permutations. O’Neill et al. [16] further developed deniability by introducing bi-deniable public-key encryption, where both sender and receiver can equivocate simultaneously without needing interaction or third parties. Their schemes, which operate in the multi-distributional model, use alternative algorithms for equivocable communication and include both simulatable and lattice-based constructions. Chi and Lei [17] introduced an audit-free cloud storage encryption scheme using deniable attribute-based encryption. Their design allows cloud storage providers to generate convincing fake user secrets, ensuring user privacy even if coercers force providers to reveal confidential data.

Dachman-Soled et al. [18] tackled a major challenge by creating a constant-round, universally composable multiparty computation protocol with adaptive security. Their protocol, relying on indistinguishability obfuscation and a common reference string, can compute any functionality while accommodating malicious adversaries. Sahai and Waters [19] introduced “punctured programs”, leveraging indistinguishability obfuscation to address various cryptographic challenges. They resolved the deniable encryption problem by allowing the sender to generate “fake” randomness and constructed several cryptographic objects, including public key encryption and non-interactive zero-knowledge proofs. De Caro et al. [20] extended deniability to functional encryption (FE), proposing models where either a master authority or the receivers themselves can generate fake keys. They provided efficient constructions for both models and showed that receiver deniability implies simulation security. Apon et al. [21] developed a flexibly bi-deniable Attribute-Based Encryption (ABE) scheme based on the Learning with Errors (LWE) assumption. Their scheme, designed for polynomial-size branching programs, advances deniable primitive construction through novel noise manipulation techniques.

Li et al. [22] proposed deniable Searchable Symmetric Encryption (Den-SSE), addressing coercion threats from both internal and external coercers. Their schemes ensure confidentiality under coercive scenarios. Cao et al. [23] introduced a new public-key encryption scheme that offers full deniability, allowing for efficient decryption of ciphertexts into different messages compared to previous schemes. Agrawal et al. [24] presented a deniable fully homomorphic encryption scheme based on LWE, achieving compact ciphertexts and public keys independent of detection probability. Their construction supports efficient online processing. Cao et al. [25] proposed authenticated deniable encryption schemes that combine deniability with user authentication. Their schemes, secure in the random oracle model, enhance efficiency and practicality for applications such as electronic voting. Coladangelo et al. [26] explored deniable encryption in the quantum realm, introducing a quantum analog of classical deniability and presenting a construction based on quantum hardness assumptions. Their work demonstrates that quantum computing enables a stronger form of deniability, termed perfect unexplainability, offering protection against preemptive coercion that is unattainable classically.

#### 1.1.3. CP-ABE in the Internet of Things

CP-ABE has become a key solution for securing data and controlling access in the Internet of Things (IoT). Li et al. [27] proposed a CP-ABE scheme with user revocation and outsourced computation, enhancing cloud storage efficiency by reducing local device overhead and mitigating collusion attacks. Li et al. [28] further introduced an ABE scheme with verifiable outsourced decryption, addressing the challenges of high decryption costs and large ciphertext sizes in resource-constrained environments while ensuring correctness for both authorized and unauthorized users. Zhang et al. [29] developed a key escrow-free CP-ABE scheme with user revocation, leveraging secure key issuance, group management, re-encryption, and outsourced decryption to address key escrow, collusion, and efficiency challenges in data sharing environments. Chen et al. [30] proposed a CP-ABE scheme with shared decryption, allowing for both independent decryption by authorized users and collaborative decryption by semi-authorized users while ensuring efficiency and honesty in decryption tasks. Guo et al. [31] introduced O3-R-CP-ABE, an efficient and revocable CP-ABE scheme for the Internet of Medical Things (IoMT), leveraging cloud servers, blockchains, and chameleon hash functions to enable fine-grained access control, fast encryption, outsourced decryption, and secure user revocation. Das et al. [32] proposed an ECC-based CP-ABE scheme tailored for resource-constrained IoT frameworks, reducing the central authority’s workload and outsourcing the decryption process to decrease user decryption overhead. Zhang et al. [33] designed a privacy-preserving, partially hidden policy CP-ABE scheme for IoT-assisted cloud computing, mitigating computational burdens on users through online decryption. Yu et al. [34] proposed a privacy protection scheme for IoT data collection, distributing security responsibilities across platforms and lowering computational costs for users through partial decryption. Li et al. [35] developed a policy-hidden multi-group CP-ABE (PH-MG-ABE) scheme to improve cloud storage security by concealing access policies and enabling flexible user group operations. Meanwhile, Chen et al. [36] proposed a revocable attribute-based encryption scheme with data integrity (RABE-DI) to enhance data security and integrity in cloud environments, addressing user revocation and improving overall system efficiency.

However, these schemes overlook the issue of coercion faced by IoT devices. When the users and data consumers of IoT devices are coerced, the confidentiality of IoT data is compromised. Additionally, with the vast volume of IoT data, fine-grained access control management for data access rights is an urgent issue that needs to be addressed.

### 1.2. Organization

The rest of this paper are organized as follows. In Section 2, we establish the necessary notation and present the mathematical tools required for the construction of the scheme. Section 3 provides the system model and formal definition of the scheme. The concrete construction is presented in Section 4. Section 5 is dedicated to the correctness for the constructed scheme. In Section 6, we analyze the deniability of the scheme’s construction. The security proof of the scheme is presented in Section 7. Section 8 presents the results of experimental simulations. The final section summarizes our work and provides an outlook on related research.

## 2. Preliminaries

In this paper, we denote [m] as {1,2,…m} and $q_{12\ldots i}$ as $q_{1}q_{2}\ldots q_{i}$. For example, $q_{123}=q_{1}q_{2}q_{3}$.

### 2.1. Negligible Function

A function ϵ: R→[0,1] is said to be negligible if and only if for ∀c≥0, there exists a constant $N_{c}\geq 0$ such that for $\forall N\geq N_{c}$, it holds that $\epsilon \left(N\right)\leq \frac{1}{N^{c}}$.

### 2.2. Prime Order Bilinear Maps

Let *q* be a large prime number, and let $G_{1}$ and $G_{2}$ be two groups of order *q*, with operations referred to as addition and multiplication, respectively. A bilinear mapping $\hat{e}$: $G_{1}\times G_{1}\to G_{2}$, satisfies the following properties:

1. Bilinearity: For any $P,Q,R\in G_{1}$, we have $\hat{e}\left(P+Q,R\right)=\hat{e}\left(P,R\right)\cdot \hat{e}\left(Q,R\right)$ and $\hat{e}\left(P,Q+R\right)=\hat{e}\left(P,Q\right)\cdot \hat{e}\left(P,R\right)$.
2. Non-degeneracy: The mapping does not map all pairs of elements from $G_{1}$ (i.e., all ordered pairs) to the identity element of $G_{2}$. Since both $G_{1}$ and $G_{2}$ are groups of prime order *q*, this implies that if *P* is a generator of $G_{1}$, then $\hat{e}\left(P,P\right)$ is a generator of $G_{2}$.
3. Computability: There exists an efficient algorithm to compute $\hat{e}\left(P,Q\right)$ for any $P,Q\in G_{1}$.

### 2.3. Composite Order Bilinear Maps

Let *G* and $\hat{G}$ be two multiplicative cyclic groups of composite order $q_{12\ldots m}$ and $q_{12\ldots m}=q_{1}q_{2}\ldots q_{m}$, where $q_{1},q_{2},\ldots ,q_{m}$ are distinct prime numbers, with a bilinear mapping *e*: $G\times G\to \hat{G}$. For each prime $q_{i}$, there exists a subgroup $G_{q_{i}}$ of *G* with an order $q_{i}$. The generators for these subgroups are denoted as $g_{1},g_{2},\ldots ,g_{m}$, respectively. Any element in the group *G* can be represented as a product $g_{1}^{a_{1}}g_{2}^{a_{2}}\ldots g_{m}^{a_{m}}$, with the exponents $a_{1},\;a_{2},\;\ldots ,\;a_{m}\in Z_{N}$. An element is considered to have no component from the subgroup $G_{q_{i}}$ if $a_{i}$ is congruent to 0 modulo $q_{i}$. An element is said to belong to the product of subgroups $\prod_{i\in S}G_{q_{i}}$, where *S* is a subset of the set {1,2,…,m}, if ∀i∈S, the corresponding $a_{i}$ is not congruent to 0 modulo $q_{i}$.

The defining characteristic of composite bilinear group structures lies in the orthogonal relationship between all constituent subgroups in relation to the bilinear pairing function *e*. This characteristic ensures that for any elements *u* belonging to subgroup $G_{q_{i}}$ and *v* belonging to a distinct subgroup $G_{q_{j}}$, where i≠j, the pairing operation yields the identity element, such that e(u,v)=1 within the group $\hat{G}$.

The security of composite group-based cryptographic systems is fundamentally grounded in the subgroupdecisionassumption. This assumption suggests that identifying whether a particular subgroup is present within a randomly chosen element of a composite order group is a computationally challenging task, unless orthogonality checks are performed. The general subgroup decision assumption is crucial for maintaining the strength and reliability of cryptographic schemes that utilize composite group architectures, which is defined as follows [37].

**Definition** **1.** *Let us define a set of non-empty subsets ${\left\{S_{i}\right\}}_{i\in [k]}$ and $S_{i}\subseteq \left[m\right]$ such that for any 2≤i≤k, it holds that $\left(S_{i}\cap S_{0}=\emptyset =S_{i}\cap S_{1}\right)\cup \left(S_{i}\cap S_{0}\neq \emptyset \neq S_{i}\cap S_{1}\right)$. With respect to a group generator G, we have the following distribution:*

$$\begin{array}{cc} \; & PP:=\left\{q_{12\ldots m},G,\hat{G},e\right\}\leftarrow_{R}GZ_{i}\leftarrow_{R}G_{S_{i}},\forall i\in 0\cup \left[k\right],\bar{Z}:=\left\{PP,Z_{2},\ldots ,Z_{k}\right\}, \end{array}$$

*then, the advantage function can be defined as:*

$$Adv_{G,A}:=\left|P\left[A\left(\bar{Z},Z_{0}\right)=1\right]-P\left[A\left(\bar{Z},Z_{1}\right)=1\right]\right|$$

*and is considered negligible for any PPT algorithm A that outputs a binary result.*

### 2.4. Discrete Logarithm Assumption

The discrete logarithm problem on a group *G* is defined as follows: Given a generator *P* of *G* and a random element *h* in *G*, compute $\log_{g}h$. This problem is believed to be difficult in many groups and is referred to as the discrete logarithm assumption on *G*.

Let GroupGen be a probabilistic polynomial–time (PPT) algorithm that, on input of a security parameter κ, outputs a description of a cyclic group *G* of order *q* (the description of *G* includes its order *q*, which is not necessarily prime, and |q|=κ) and a generator g∈G. The discrete logarithm assumption is defined as follows.

**Definition** **2.** *If for all PPT algorithms A, the following expression is negligible:*

(1) $$\begin{array}{c} Pr[\left(G,g\right)\leftarrow GroupGen\left(\kappa \right);h\leftarrow_{R}G;x\leftarrow A\left(G,g,h\right)s.t.g^{x}=h], \end{array}$$

*then, the discrete logarithm problem of GroupGen is hard.*

### 2.5. Linear Secret Sharing Scheme

Let *F* be a finite field and S⊆F be a set of secrets. A secret sharing scheme Π over *F* is said to be linear if it satisfies the following two conditions [38]:

1. Each participant’s share is a vector over *F*.
2. For every authorized set of participants, the function to reconstruct the secret is linear. Formally, the share-generating matrix *A* in an LSSS scheme Π is a t×k matrix. Each row of *M*, indexed by i=1,…,t, corresponds to a party labeled by the mapping function f(i) from {1,…,t} to party field *P*. When we have a column vector $\vec{\mu }=\left(s,s_{2},\ldots ,s_{k}\right)$, where *s* is the secret to be shared and $s_{2},\ldots ,s_{k}\in Z_{p}$ are randomly chosen, the vector M·μ represents the *t* shares of *s* according to Π. The share ${\left(M\cdot \vec{\mu }\right)}_{i}$ is assigned to party f(i).

The property of linear reconstruction in an LSSS scheme is characterized as follows: Given an authorized subset of attributes *S*, we define a set *T* as the collection of indices T={i:f(i)∈S}, which is a subset of {1,…,t}. It then follows that the vector $\left(1,0,\ldots ,0\right)\in Z^{k}$ is included in the span of the set of vectors $\left\{M_{i}\right\}$, where i∈T. This indicates the existence of a set of constants ${\left\{\theta_{i}\right\}}_{i\in T}$ such that $\sum_{i\in T}\theta_{i}\cdot M_{i}=\left(1,0,\ldots ,0\right)$. As a result, it holds that $\sum_{i\in T}\theta_{i}\cdot M_{i}\cdot \mu =s$.

In the work presented by [6], an access structure (M,f) is associated with a boolean expression *T*, and similarly, a revoked access structure $\left(\tilde{M},\tilde{f}\right)$ is linked to a boolean expression $\tilde{T}$. The resulting revoked access policy $T^{\prime }$ for the policy $\left(M^{\prime },f^{\prime }\right)$ is formulated as $T^{\prime }=\left(T\;⋀\;\tilde{T}\right)$.

### 2.6. Chameleon Hash

The chameleon hash scheme, first introduced in [39], is akin to conventional secure hash functions in that it upholds two principal attributes: collisionresistance and semanticsecurity. Beyond these, it also facilitates collisionforgery through the use of a pre-established trapdoor. A chameleon hash function takes two primary components as input: the message *m* and a random string *r*, the latter of which enables the adjustment of the hash output to fit the desired message. The following outlines the specifications for the three aforementioned properties: collisionresistance, semanticsecurity, and collisionforgery.

**Definition** **3** (Collision resistance)**.** *In the chameleon hash scheme {PK,SK,CH(·,·)}, the public key PK is openly available, the secret key SK serves as the trapdoor, and CH(·,·) represents the hash function itself. The concept of collision resistance is characterized by the computational difficulty faced by any algorithm A in finding a random string $\hat{r}$ that satisfies the equation $CH\left(m,r\right)=CH\left(\hat{m},\hat{r}\right)$ for two distinct messages m and $\hat{m}$, without the benefit of the trapdoor key SK.*

**Definition** **4** (Semantic security)**.** *In the chameleon hash scheme {PK,SK,CH(·,·)}, the public key PK is accessible to all, the trapdoor key SK is kept private, and CH(·,·) is the underlying hash function. Semantic security in this context is defined by the infeasibility for any computational algorithm to discern between the output distributions of CH(m,r) and $CH(\hat{m},\hat{r})$ for any given pair of messages m and $\hat{m}$, along with their corresponding random strings r and $\hat{r}$.*

**Definition** **5** (Collision forgery)**.** *In the chameleon hash scheme, which is defined by the triples {PK,SK,CH(·,·)}, the public key PK is openly disclosed, the trapdoor SK is maintained as confidential, and the hash function is represented by CH(·,·). We define a collision forgery scheme as a scenario where there exists at least one PPT algorithm A that, when provided with SK, outputs a string $\hat{r}$ such that $CH\left(m,r\right)=CH\left(\hat{m},\hat{r}\right)$, where m and $\hat{m}$ are two distinct messages and r is a random string.*

In this paper, we refer to the public information associated with the chameleon hash as CH and the operational function of the chameleon hash as CH(·,·).

## 3. System Model and Formal Definition

In this section, we present the system model and formal definition of our scheme, which consists of three entities and nine algorithms. The responsibilities of the entities and the functionalities of the algorithms are as follows.

### 3.1. System Model

**Figure 1:**
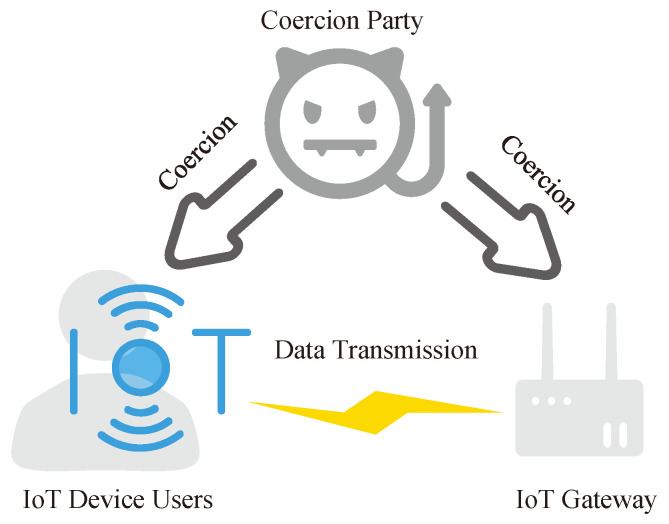
System model.

Our scheme is primarily applied at the network layer and application layer of the IoT architecture. It involves three types of entities: IoT device users, IoT gateways, and coercers. The system model of the scheme is shown in Figure 1.

- IoT device users: IoT device users are the data owners in the IoT, who collect real-world data through IoT devices. For example, they may use smart wearable devices to gather health data about individuals.
- IoT gateways: IoT gateways are the processors of IoT data. They receive the data collected by IoT device users and process and analyze them, further facilitating resource scheduling for IoT devices or providing processed/computed data for their subsequent use.
- Coercers: The coercive party refers to corrupted authoritative institutions or other malicious entities. They compel IoT device users or IoT gateways to disclose their private keys or raw data through coercive means.

### 3.2. Formal Definition

We describe the executing entities and functions of the algorithms within the scheme, taking into account the entities involved in the scheme.

First, in the system initialization phase, algorithms SetupAlgo1(·) and SetupAlgo2(·) are executed to generate the necessary parameters for system operation. These include the global parameter GP, which is used in all algorithms during the subsequent data transmission and reception phases; the coercion-resistance parameter CRP, which is used in the EncAlgo2 algorithm of the data transmission phase to generate coercion-resistant ciphertext; the master secret key MSK, which is used by the KeyGenAlgo1 algorithm in the system user key generation phase to generate the real user secret key; and the coercion-resistance secret key CRK, which is used by the KeyGenAlgo2 algorithm in the system user key generation phase to generate the fake user secret key for coercion resistance.

- SetupAlgo1(λ,U)→(GP,MSK): This algorithm is performed by IoT gateways, which inputs a security parameter λ and a set of all possible attributes *U*, outputs a global parameter GP, which is public, and outputs a master secret key MSK, which maintains privacy.
- SetupAlgo2(λ,U)→(GP,CRP,CRK): This algorithm is also performed by IoT gateways, which inputs a security parameter λ and a set of all possible attributes *U*, and in addition to output GP and MSK, the algorithm also outputs a coercion-resistance parameter CRP and a coercion-resistance secret key CRK, which are kept secret from users outside the system.

Next is the system user key generation phase, which includes two algorithms (KeyGenAlgo1(·) and KeyGenAlgo2(·)), where algorithm KeyGenAlgo1(·) generates the user’s real secret key RSK and algorithm KeyGenAlgo2(·) generates the fake secret key FSK used for coercion resistance.

- KeyGenAlgo1(MSK,S)→RSK: This algorithm is also performed by IoT gateways, which inputs the master secret key MSK and the specified set of attributes *S* and S⊆U, outputting a real secret key RSK related to *S*.
- KeyGenAlgo2(CRK,S)→(RSK,FSK): This algorithm is also performed by IoT gateways, which inputs MSK and *S*, outputting RSK and a fake secret key FSK.

Following this is the data transmission phase, which consists of algorithms EncAlgo1(·) and EncAlgo2(·). Algorithm EncAlgo1(·) generates normal ciphertext NC without coercion resistance, while algorithm EncAlgo2(·) generates coercion-resistant ciphertext CRC.

- EncAlgo1(GP,M,(A,f))→NC: This algorithm is performed by IoT device users, which inputs GP, a message *M*, and an access policy (A,f), outputting a normal ciphertext NC.
- $EncAlgo2(GP,CRP,M,\hat{M},\left(A,f\right))\to CRC$: This algorithm is also performed by IoT device users, which inputs GP, DP, *M*, a fake message $\hat{M}$, and (A,f), outputting a coercion-resistant ciphertext CRC.

The next phase is the policy revocation phase, where the algorithm does not distinguish between the types of input ciphertext but outputs a revoked ciphertext $C^{\prime }$. However, the algorithm inherits the properties of the input ciphertext: if the input ciphertext is a normal one without coercion resistance, the corresponding revoked ciphertext will also lack coercion resistance; otherwise, if the input ciphertext is coercion-resistant, the revoked ciphertext will also retain coercion resistance.

- $RevokeAlgo\left(NC/CRC,\left(\tilde{A},\tilde{f}\right)\right)\to NC^{\prime }/CRC^{\prime }$: This algorithm is performed by IoT gateways, which inputs NC/CRC and a revoked access policy matrix $\left(\tilde{A},\tilde{f}\right)$, outputting a revoked ciphertext $NC^{\prime }/CRC^{\prime }$.

Finally, the data reception phase consists of two algorithms: DecAlgo1(·) and DecAlgo2(·). The first algorithm, DecAlgo1(·), is used to decrypt the original ciphertext that has not been revoked by the algorithm RevokeAlgo(·). The second algorithm, DecAlgo2(·), is used to decrypt the revoked ciphertext generated by the revoke algorithm.

- $DecAlgo1\left(GP,RSK/FSK,NC/CRC\right)\to \left\{M,\hat{M},⊥\right\}$: This algorithm, which can be performed by either IoT gateways or the coercer, takes in GP, RSK/FSK, and NC/CRC as inputs. If the input is RSK, it outputs the real message *M*. If the input is FSK, it outputs the fake message $\hat{M}$. For any other input, it returns an error symbol ⊥.
- $DecAlgo2\left(GP,RSK/FSK,NC/CRC,NC^{\prime }/CRC^{\prime }\right)\to \left\{M,\hat{M},⊥\right\}$: This algorithm, which can also be executed by either IoT gateways or the coercer, takes in GP, RSK/FSK, NC/CRC, and $NC^{\prime }/CRC^{\prime }$ as inputs. Similarly, if the input is RSK, it outputs the real message *M*. If the input is FSK, it outputs the fake message $\hat{M}$. For any other input, it returns an error symbol ⊥.

In this definition, we require algorithms SetupAlgo2, KeyGenAlgo2, and EncAlgo2 to be private. That is, for users outside the system, including coercers, there are only six algorithms: SetupAlgo1, KeyGenAlgo1, EncAlgo1, RevokeAlgo1, DecAlgo1, DecAlgo2.

We also require that the definition satisfies the following properties:

- Semantic security: This scheme is a semantically secure CP-ABE scheme. The security model adopted in this paper is identical to that of Waters’ scheme [40]. The semantic security of this work is established by a reduction to the semantic security of Waters’ scheme. For the sake of brevity, the detailed security model is not repeated here. Readers are referred to [40] for a complete formalization.
- Ciphertext consistency: The four types of ciphertexts (normal ciphertext NC, coercion-resistant ciphertext CRC, revoked ciphertext $NC^{\prime }$, and coercion-resistant and revoked ciphertext $CRC^{\prime }$) generated by the scheme are computationally indistinguishable.
- Key consistency: The two types of secret keys (real secret key RSK and fake secret key FSK) generated by this scheme are computationally indistinguishable.
- Coercion resistance: If the scheme satisfies the properties of ciphertext consistency and key consistency, it can achieve coercion resistance.

## 4. Construction

In this section, based on the definition in the previous section, we give a concrete construction based on the composite order bilinear map and chameleon hash function. We achieve efficient attribute revocation by utilizing the same revocation matrix as in [13]. The detailed scheme construction is given below.

First, the detailed construction of the system initialization phase is described.

- SetupAlgo1(λ,U)→(GP,MSK): This algorithm generates a tuple $(q_{1},q_{2},q_{3},q_{123},G_{q_{1}},$$G_{q_{3}},G,G_{T},e)$, where $q_{1}$, $q_{2}$, and $q_{3}$ represent distinct prime numbers, $q_{123}=q_{1}q_{2}q_{3}$, $G_{q_{1}}$, $G_{q_{3}}$, *G*, and $G_{T}$ denote the groups with order $q_{1}$, $q_{3}$, $q_{123}$ and $q_{123}$, respectively, and *e* is a bilinear map function: $G\times G\to G_{T}$. Then, this algorithm selects generators $g_{1}$ of $G_{q_{1}}$ and $g_{3}$ of $G_{q_{3}}$, and it randomly picks *a* and α from $Z_{n}^{*}$. In addition, this algorithm chooses a hash function $\bar{H}:\left\{0,1\right\}\to G_{q_{3}}$. Finally, we set the master secret key $MSK=g_{13}^{\alpha }$ and the public global parameter $GP=\{e,g_{13},g_{123},g_{13}^{a},e{\left(g_{13},g_{13}\right)}^{\alpha },G,G_{T},\bar{H}\}$.
- SetupAlgo2(λ,U)→(GP,CRP,CRK): This algorithm runs SetupAlgo1 algorithm to obtain GP. Then, this algorithm selects generator $g_{2}$ of $G_{q_{2}}$ and outputs the coercion-resistant public parameter $CRP=\{g_{23},g_{23}^{a},e{\left(g_{3},g_{3}\right)}^{\alpha },e{\left(g_{23},g_{23}\right)}^{\alpha }\}$ and the coercion-resistant master secret key $CRK=\{g_{13}^{\alpha },g_{123},g_{123}^{\alpha }\}$.

Next, the detailed construction of the system user key generation phase is presented as follows.

- KeyGenAlgo1(MSK,S)→RSK: This algorithm randomly selects $t\in Z_{q_{123}}$ and outputs the real secret key:
  $$RSK=\{S,RK,RL,{\left\{RK_{x}\right\}}_{\forall x\in S}\},$$
  where *S* is the given attribute set, $RK=g_{13}^{\alpha +at}$, $RL=g_{13}^{t}$, and $RK_{x}=\bar{H}{\left(x\right)}^{t}$.
- KeyGenAlgo2(CRK,S)→(RSK,FSK): This algorithm runs KeyGenAlgo1 algorithm to obtain RSK bounds to *S*. Then, the algorithm randomly chooses $\hat{t}\in Z_{q_{123}}$ and outputs the fake secret key:
  $$FSK=\{S,FK,FL,{\left\{FK_{x}\right\}}_{\forall x\in S}\},$$
  where *S* is the same attribute set as in RSK, $FK=g_{123}^{\alpha +a\hat{t}}$, $FL=g_{123}^{\hat{t}}$ and $FK_{x}=\bar{H}{\left(x\right)}^{\hat{t}}$.

Next, the detailed construction of the algorithms involved in the data transmission phase is presented as follows.

- EncAlgo1(GP,M,(A,f))→NC: For a given message *M* and a specified access policy (A,f), where *A* is an m×n matrix and *f* can map each row of *A* to a corresponding attribute $A_{i}$ denoting the *i*th row of *A*, this algorithm then randomly selects a vector $\vec{\mu }=\left(s,s_{1},\ldots ,s_{n-1}\right)\in Z_{q_{123}}^{n}$ and computes $\lambda_{i}=A_{i}\cdot \vec{\mu }$, where i∈[1,m]. Then, the algorithm randomly chooses $r_{i}\in Z_{q_{123}}$, where i∈[1,m]. This algorithm continues to select a one-way hash function H(·,·) with two inputs. Note that *H* is determined during encryption; thus, each transaction can use a different *H*. Finally, this algorithm needs to flip two coins, $b_{0}$ and $b_{1}$, and select two random strings, $d_{0}$ and $d_{1}$. It outputs the normal ciphertext NC:
  $$\begin{array}{c} NC=\{\left(A,f\right),NC_{0},NC_{1},ND,\left(NE_{1},NF_{1}\right),\ldots ,\left(NE_{m},NF_{m}\right),H,d_{0},d_{1},h\}, \end{array}$$
  where
  $$\begin{array}{cc} \; & NC_{b_{0}}=M\cdot e{\left(g_{13},g_{13}\right)}^{\alpha s},NC_{1-b_{0}}\overset{R}{\leftarrow }G_{T},ND=g_{13}^{s},\\ \; & NE_{i}=g_{13}^{a\lambda_{i}}\bar{H}{\left(f\left(i\right)\right)}^{-r_{i}},NF_{i}=g_{13}^{r_{i}},\forall i\in \left[1,m\right],\\ \; & h=H\left(M,d_{b_{1}}\right),h\neq H\left(C_{1-b_{0}}\cdot e{\left(g_{13},g_{13}\right)}^{-\alpha s},d_{1-b_{1}}\right). \end{array}$$
- $EncAlgo2(GP,CRP,M,\hat{M},\left(A,f\right))\to CRC$: Similar to the EncAlgo1 algorithm, this algorithm randomly chooses $\lambda_{i}$, where i∈[1,m]. In particular, the algorithm chooses the chameleon hash function CH instead of the one-way hash function. Note that without the trapdoor, the chameleon hash function is the same as the normal one-way hash function. Thus, the sender can claim that the function is a standard two-input one-way hash function. It outputs the coercion-resistant ciphertext CRC:
  $$\begin{array}{cc} CRC= & \{\left(A,f\right),CRC_{0},CRC_{1},CRD,\left(CRE_{1},CRF_{1}\right),\ldots ,\left(CRE_{m},CRF_{m}\right\},CH,d_{0},d_{1},\hat{h}), \end{array}$$
  where
  $$\begin{array}{cc} \; & CRC_{b_{0}}=M\cdot e{\left(g_{3},g_{3}\right)}^{\alpha s},CRC_{1-b_{0}}=\hat{M}\cdot e{\left(g_{23},g_{23}\right)}^{\alpha s},CRD=g_{23}^{s},\\ \; & CRE_{i}=g_{23}^{a\lambda_{i}}\bar{H}{\left(f\left(i\right)\right)}^{-r_{i}},CRF_{i}=g_{13}^{r_{i}},\forall i\in \left[1,m\right],\\ \; & \hat{h}=CH\left(M,d_{b_{1}}\right)=CH\left(\hat{M},d_{1-b_{1}}\right). \end{array}$$

Next, the algorithm implementation for the policy revocation phase is described. As mentioned earlier, this phase includes only one algorithm, and its construction details are as follows.

- $RevokeAlgo\left(NC/CRC,\left(\tilde{A},\tilde{f}\right)\right)\to NC^{\prime }/CRC^{\prime }$: Input a ciphertext NC/CRC and a revocation access policy $\left(\tilde{A},\tilde{f}\right)$, where *A* and $\tilde{A}$ are m×n and $\tilde{m}\times \tilde{n}$ matrixes, outputting a revoked ciphertext for access policy $\left(A^{\prime },f^{\prime }\right)$. Set $\left(A^{\prime },f^{\prime }\right)$ as follows:
  (2) A′=A−a1|00A˜, f′=f(i)i≤mf˜(i−l)i≥m,
  where $a_{1}$ is the first column of *A*. Note that $A^{\prime }$ is an $m^{\prime }\times n^{\prime }$ matrix, where $m^{\prime }=m+\tilde{m}$ and $n^{\prime }=n+\tilde{n}$.
  The renovation process can also be divided into the following two types:
  – For the normal ciphertext NC, set $NC_{0}^{\prime \prime }=NC_{0}$, $NC_{1}^{\prime \prime }=NC_{1}$, $ND^{\prime \prime }=ND$,
    $$\left\{\begin{array}{cc} NE_{i}^{\prime \prime }=NE_{i},NF_{i}^{\prime \prime }=NF_{i} & i\in \left[1,m\right]\\ NE_{i}^{\prime \prime }=1_{G_{T}},NF_{i}^{\prime \prime }=1_{G_{T}} & i\in \left[1+m,m^{\prime }\right] \end{array}\right.,$$
    where $1_{G_{T}}$ is the identity element of group $G_{T}$.
    Next, select a random vector ${\vec{\mu }}^{\prime \prime \prime }=\left(s^{\prime \prime \prime },s_{1}^{\prime \prime \prime },\ldots ,s_{n^{\prime }-1}^{\prime \prime \prime }\right)\in Z_{q_{123}}^{n^{\prime }}$ and compute $\lambda_{i}^{\prime \prime \prime }=A_{i}^{\prime }\cdot {\vec{\mu }}^{\prime \prime \prime }$, where $i\in [1,n^{\prime }]$. Then, for each $i\in [1,n^{\prime }]$, choose randomly $r_{i}^{\prime \prime \prime }\in Z_{q_{123}}$ and compute a random ciphertext $NC^{\prime \prime \prime }$ as
    $$\begin{array}{cc} \; & NC_{b_{0}}^{\prime \prime \prime }=e{\left(g_{13},g_{13}\right)}^{\alpha s^{\prime \prime \prime }},NC_{1-b_{0}}^{\prime \prime \prime }\overset{R}{\leftarrow }G_{q_{13}},ND^{\prime \prime \prime }=g_{13}^{s^{\prime \prime \prime }},\\ \; & NE_{i}^{\prime \prime \prime }={\left(g_{13}\right)}^{a\lambda_{i}^{\prime \prime \prime }}\bar{H}{\left(f\left(i\right)\right)}^{-r_{i}^{\prime \prime \prime }},NF_{i}^{\prime \prime \prime }={\left(g_{13}\right)}^{r_{i}^{\prime \prime \prime }},\forall i\in \left[1,m^{\prime }\right]. \end{array}$$
    Then, compute the following:
    $$\begin{array}{cc} \; & NC_{b_{0}}^{\prime }=NC_{b_{0}}^{\prime \prime }\cdot NC_{b_{0}}^{\prime \prime \prime },NC_{1-b_{0}}^{\prime }=NC_{1-b_{0}}^{\prime \prime }\cdot NC_{1-b_{0}}^{\prime \prime \prime },ND^{\prime }=ND^{\prime \prime }\cdot ND^{\prime \prime \prime },\\ \; & NE_{i}^{\prime }=NE_{i}^{\prime \prime }\cdot NE_{i}^{\prime \prime \prime },NF_{i}^{\prime }=NF_{i}^{\prime \prime }\cdot NF_{i}^{\prime \prime \prime },\forall i\in \left[1,m^{\prime }\right]. \end{array}$$
    Finally, set $h^{\prime }=h$ and return the revoked ciphertext
    $$\begin{array}{c} NC^{\prime }=\left\{\left(A^{\prime },f^{\prime }\right),NC_{0}^{\prime },NC_{1}^{\prime },ND^{\prime },\left(NE_{1}^{\prime },NF_{1}^{\prime }\right),\ldots ,\left(NE_{m^{\prime }}^{\prime },NF_{m^{\prime }}^{\prime }\right),H,d_{0},d_{1},h^{\prime }\right\}. \end{array}$$
  – Similarly, for the coercion-resistant ciphertext CRC, set $CRC_{0}^{\prime \prime }=CRC_{0}$, $CRC_{1}^{\prime \prime }=CRC_{1}$, $CRD^{\prime \prime }=CRD$,
    CREi″=CREi,CRFi″=CRFii∈[1,m]CREi″=1GT,CRFi″=1GTi∈[1+m,m′],
    where $1_{G_{T}}$ is the identity element of group $G_{T}$.
    Then, select a random vector ${\vec{\hat{\mu }}}^{\prime \prime \prime }=\left({\hat{s}}^{\prime \prime \prime },{\hat{s}}_{1}^{\prime \prime \prime },\ldots ,{\hat{s}}_{n-1}^{\prime \prime \prime }\right)\in Z_{q_{123}}^{n^{\prime }}$ and compute ${\hat{\lambda }}_{i}^{\prime \prime \prime }=A_{i}^{\prime }\cdot {\vec{\hat{\mu }}}^{\prime \prime \prime }$, where $i\in [1,m^{\prime }]$. Choose randomly ${\hat{r}}_{i}^{\prime \prime \prime }\in Z_{q_{123}}$ for each $i\in [1,m^{\prime }]$, then compute a random ciphertext $CRC^{\prime \prime \prime }$ as follows:
    $$\begin{array}{cc} \; & CRC_{b_{0}}^{\prime \prime \prime }=e{\left(g_{13},g_{13}\right)}^{\alpha {\hat{s}}^{\prime \prime \prime }},CRC_{1-b_{0}}^{\prime \prime \prime }\overset{R}{\leftarrow }G_{q_{13}},CRD^{\prime \prime \prime }=g_{13}^{{\hat{s}}^{\prime \prime \prime }},\\ \; & CRE_{i}^{\prime \prime \prime }={\left(g_{13}\right)}^{a{\hat{\lambda }}_{i}^{\prime \prime \prime }}\bar{H}{\left(f\left(i\right)\right)}^{-{\hat{r}}_{i}^{\prime \prime \prime }},CRF_{i}^{\prime \prime \prime }={\left(g_{13}\right)}^{{\hat{r}}_{i}^{\prime \prime \prime }},\forall i\in \left[1,m^{\prime }\right]. \end{array}$$
    Next, compute the following:
    $$\begin{array}{cc} \; & CRC_{b_{0}}^{\prime }=CRC_{b_{0}}^{\prime \prime }\cdot CRC_{b_{0}}^{\prime \prime \prime },CRC_{1-b_{0}}^{\prime }=CRC_{1-b_{0}}^{\prime \prime }\cdot CRC_{1-b_{0}}^{\prime \prime \prime },CRD^{\prime }=CRD^{\prime \prime }\cdot CRD^{\prime \prime \prime },\\ \; & CRE_{i}^{\prime }=CRE_{i}^{\prime \prime }\cdot CRE_{i}^{\prime \prime \prime },CRF_{i}^{\prime }=CRF_{i}^{\prime \prime }\cdot CRF_{i}^{\prime \prime \prime },\forall i\in \left[1,m^{\prime }\right]. \end{array}$$
    Finally, set ${\hat{h}}^{\prime }=\hat{h}$ and return the coercion-resistant and revoked ciphertext
    $$\begin{array}{cc} CRC^{\prime }= & \{\left(A^{\prime },f^{\prime }\right),CRC_{0}^{\prime },CRC_{1}^{\prime },CRD^{\prime },\left(CRE_{1}^{\prime },CRF_{1}^{\prime }\right),\ldots ,\left(CRE_{m^{\prime }}^{\prime },CRF_{m^{\prime }}^{\prime }\right),CH,\\ \; & d_{0},d_{1},{\hat{h}}^{\prime }\}. \end{array}$$

Finally, the construction details of the two algorithms in the data reception phase are as follows. For coerced system users, the fake secret key FSK will be used to decrypt the data; otherwise, the real secret key RSK will be used.

- $DecAlgo1\left(GP,RSK/FSK,NC/CRC\right)\to \left\{M,\hat{M},⊥\right\}$: The receiver first verifies that R(S,(A,f)). If R(S,(A,f))≠1, output ⊥. Otherwise, find the set T={i:f(i)∈S}⊂{1,…,m}. Then, this algorithm finds a set of constants $\omega \in Z_{q_{123}}$ such that $\sum_{i\in T}A_{i}\cdot \omega_{i}=\left(1,0,\ldots ,0\right)$.
  The decryption process can be divided into the following two types:
  – The message is encrypted by the EncAlgo1 algorithm, then compute the following:
    $$m_{\left\{0,1\right\}}=NC_{\left\{0,1\right\}}\cdot \frac{\prod_{i\in T}{\left(e\left(NE_{i},L\right)\cdot e\left(NF_{i},K_{f(i)}\right)\right)}^{\omega_{i}}}{e(ND,K)},$$
    where L∈{RL,FL}, $K_{f(i)}\in \left\{RK_{f(i)},FK_{f(i)}\right\}$, and K∈{RK,FK}. Finally, this algorithm calculates
    $$h_{i,j}=H\left(m_{i},d_{j}\right),\forall i,j\in \left\{0,1\right\}.$$
    If $h_{i,j}=h$, then $m_{i}=M$ is the true message and is returned; Otherwise, return ⊥.
  – The message is encrypted by the EncAlgo2 algorithm, then compute the following:
    $$m_{\left\{0,1\right\}}=CRC_{\left\{0,1\right\}}\cdot \frac{\prod_{i\in T}{\left(e\left(CRE_{i},L\right)\cdot e\left(CRF_{i},K_{f(i)}\right)\right)}^{\omega_{i}}}{e(CRD,K)},$$
    where L∈{RL,FL}, $K_{f(i)}\in \left\{RK_{f(i)},FK_{f(i)}\right\}$, and K∈{RK,FK}. Finally, this algorithm calculates the following:
    $$h_{i,j}=CH\left(m_{i},t_{j}\right),\forall i,j\in \left\{0,1\right\}.$$
    If $h_{i,j}=h$, then $m_{i}=M$ is the true message and is returned; And if $h_{i,j}=\hat{h}$, then $m_{i}=\hat{M}$ is the fake message and is returned. Otherwise, this algorithm returns ⊥.
- $DecAlgo2\left(GP,RSK/FSK,NC/CRC,NC^{\prime }/CRC^{\prime }\right)\to \left\{M,\hat{M},⊥\right\}$: Input a secret key RSK/FSK that corresponds to a specific attribute set *S*, the initial ciphertext NC/CRC, and its revoked counterpart $NC^{\prime }/CRC^{\prime }$. Now, we still discuss the output according to the type of ciphertext.
  – For the normal ciphertext NC and its revoked counterpart $NC^{\prime }$, this algorithm first validates whether $h^{\prime }=h$. If $h^{\prime }\neq h$, it outputs an error symbol ⊥ and terminates. Then, it verifies the condition $R(S,\left(A^{\prime },f^{\prime }\right))=1$. If $R(S,\left(A^{\prime },f^{\prime }\right))\neq 1$, the algorithm again outputs ⊥ and terminates. If the above two conditions are both satisfied, the algorithm proceeds to identify the subset $T^{\prime }$ of indices $\left\{1,\ldots ,m^{\prime }\right\}$, where $T^{\prime }=\left\{i:f^{\prime }\left(i\right)\in S\right\}$. Furthermore, the algorithm finds a set of integers $\omega^{\prime }\in Z_{q_{123}}$ that fulfill the equation $\sum_{i\in T^{\prime }}A_{i}^{\prime }\cdot \omega_{i}^{\prime }=\left(1,0,\ldots ,0\right)$. This algorithm computes
    $$m_{\left\{0,1\right\}}=NC_{\left\{0,1\right\}}^{\prime }\cdot \frac{\prod_{i\in T^{\prime }}{\left(e\left(NE_{i}^{\prime },L\right)\cdot e\left(NF_{i}^{\prime },K_{f^{\prime }\left(i\right)}\right)\right)}^{\omega_{i}^{\prime }}}{e(ND^{\prime },K)},$$
    where L∈{RL,FL}, $K_{f(i)}\in \left\{RK_{f(i)},FK_{f(i)}\right\}$, and K∈{RK,FK}. Finally, this algorithm calculates
    $$h_{i,j}=H\left(m_{i},d_{j}\right),\forall i,j\in \left\{0,1\right\}.$$
    If $h_{i,j}=h$, then $m_{i}=M$ is the true message and is returned; Otherwise, return ⊥.
  – Similarly, for the coercion-resistant ciphertext CRC and its revoked counterpart $CRC^{\prime }$, this algorithm first validates whether $\left({\hat{h}}^{\prime }=\hat{h}\right)\cap \left(R\left(S,\left(A^{\prime },f^{\prime }\right)\right)=1\right)$. If this condition is not met, the algorithm outputs ⊥ and terminates. Otherwise, the algorithm proceeds to identify the subset $T^{\prime }$ of indices $\left\{1,\ldots ,m^{\prime }\right\}$, where $T^{\prime }=\left\{i:f^{\prime }\left(i\right)\in S\right\}$. Then, the algorithm finds a set of integers ${\hat{\omega }}^{\prime }\in Z_{q_{123}}$ that fulfill the equation $\sum_{i\in T^{\prime }}A_{i}^{\prime }\cdot {\hat{\omega }}_{i}^{\prime }=\left(1,0,\ldots ,0\right)$. This algorithm computes
    $$m_{\left\{0,1\right\}}=CRC_{\left\{0,1\right\}}^{\prime }\cdot \frac{\prod_{i\in T^{\prime }}{\left(e\left(CRE_{i}^{\prime },L\right)\cdot e\left(CRF_{i}^{\prime },K_{f^{\prime }\left(i\right)}\right)\right)}^{\omega_{i}^{\prime }}}{e(CRD^{\prime },K)},$$
    where L∈{RL,FL}, $K_{f(i)}\in \left\{RK_{f(i)},FK_{f(i)}\right\}$, and K∈{RK,FK}. Finally, this algorithm calculates
    $$h_{i,j}=CH\left(m_{i},d_{j}\right),\forall i,j\in \left\{0,1\right\}.$$
    If $h_{i,j}=h$, then $m_{i}=M$ is the true message and is returned; And if $h_{i,j}=\hat{h}$, then $m_{i}=\hat{M}$ is the fake message and is returned. Otherwise, this algorithm returns ⊥.

## 5. Correctness

In the scheme construction of this paper, there are four types of ciphertext: the normal ciphertext NC, the coercion-resistant ciphertext CRC, the revoked ciphertext $NC^{\prime }$, and the coercion-resistant and revoked ciphertext $CRC^{\prime }$, an. There are two types of secret keys: the real secret key RSK and the fake secret key FSK. In this section, we prove that the results of the decryption of the four ciphertexts by the two keys are expected.

- Use RSK to decrypt NC. We have the following:
  $$\begin{array}{cc} \; & \frac{\prod_{i\in T}{\left(e\left(NE_{i},RL\right)\cdot e\left(NF_{i},RK_{f(i)}\right)\right)}^{\omega_{i}}}{e(ND,RK)}\\ \; & =\frac{\prod_{i\in T}{\left(e\left(g_{13}^{a\lambda_{i}},g_{13}^{t}\right)\right)}^{\omega_{i}}}{e(g_{13}^{s},g_{13}^{\alpha +at})}\\ \; & =\frac{e{\left(g_{13},g_{13}\right)}^{at\sum_{i\in T}\lambda_{i}\omega_{i}}}{e{\left(g_{13},g_{13}\right)}^{s(\alpha +at)}}\\ \; & =e{\left(g_{13},g_{13}\right)}^{-\alpha s}, \end{array}$$
  where $e\left(NE_{i},RL\right)=e\left(g_{13}^{a\lambda_{i}}\bar{H}{\left(f\left(i\right)\right)}^{-r_{i}},g_{13}^{t}\right)$ and $e\left(NF_{i},RK_{f(i)}\right)=e\left(g_{13}^{r_{i}},\bar{H}{\left(f\left(i\right)\right)}^{t}\right).$
  Thus, we have
  $$\begin{array}{c} NC_{b_{0}}\cdot \frac{\prod_{i\in T}{\left(e\left(NE_{i},RL\right)\cdot e\left(NF_{i},RK_{f(i)}\right)\right)}^{\omega_{i}}}{e(ND,RK)}=M. \end{array}$$
- Use RSK to decrypt CRC. We have the following:
  $$\begin{array}{cc} \; & \frac{\prod_{i\in T}{\left(e\left(CRE_{i},RL\right)\cdot e\left(CRF_{i},RK_{f(i)}\right)\right)}^{\omega_{i}}}{e(CRD,RK)}\\ \; & =\frac{\prod_{i\in T}{\left(e\left(g_{23}^{a\lambda_{i}},g_{13}^{t}\right)\right)}^{\omega_{i}}}{e(g_{23}^{s},g_{13}^{\alpha +at})}\\ \; & =\frac{e{\left(g_{23},g_{13}\right)}^{at\sum_{i\in T}\lambda_{i}\omega_{i}}}{e{\left(g_{23},g_{13}\right)}^{s(\alpha +at)}}\\ \; & =e{\left(g_{3},g_{3}\right)}^{-\alpha s}, \end{array}$$
  where $e\left(CRE_{i},RL\right)=e\left(g_{23}^{a\lambda_{i}}\bar{H}{\left(f\left(i\right)\right)}^{-r_{i}},g_{13}^{t}\right)$ and $e\left(CRF_{i},RK_{f(i)}\right)=e\left(g_{13}^{r_{i}},\bar{H}{\left(f\left(i\right)\right)}^{t}\right)$.
  Thus, we have the following:
  $$\begin{array}{c} CRC_{b_{0}}\cdot \frac{\prod_{i\in T}{\left(e\left(CRE_{i},RL\right)\cdot e\left(CRF_{i},RK_{f(i)}\right)\right)}^{\omega_{i}}}{e(CRD,RK)}=M. \end{array}$$
- Use RSK to decrypt $NC^{\prime }$. We have the following:
  $$\begin{array}{cc} \; & \frac{\prod_{i\in T}{\left(e\left(NE_{i}^{\prime },RL\right)\cdot e\left(NF_{i}^{\prime },RK_{f(i)}\right)\right)}^{\omega_{i}^{\prime }}}{e(ND^{\prime },RK)}\\ \; & =\frac{\prod_{i\in T_{1}^{\prime }}{\left(e\left(g_{13}^{a(\lambda_{i}+\lambda_{i}^{\prime \prime \prime })},g_{13}^{t}\right)\right)}^{\omega_{i}^{\prime }}}{e(g_{13}^{s+s^{\prime \prime \prime }},g_{13}^{\alpha +at})}+\frac{\prod_{i\in T_{2}^{\prime }}\left(\bar{H}{\left(f\left(i\right)\right)}^{-r_{i}^{\prime \prime \prime }}\;\;,g_{13}^{t}\right)\;\cdot \;e\left(g_{13}^{r_{i}^{\prime \prime \prime }},\bar{H}{\left(f\left(i\right)\right)}^{t}\right){)}^{\omega_{i}^{\prime }}}{e(g_{13}^{s+s^{\prime \prime \prime }}\;\;,g_{13}^{\alpha +at})}\\ \; & =\frac{\prod_{i\in T_{1}^{\prime }}{\left(e{\left(g_{13},g_{13}\right)}^{at(\lambda_{i}+\lambda_{i}^{\prime \prime \prime })}\right)}^{\omega_{i}^{\prime }}}{e{\left(g_{13},g_{13}\right)}^{\left(\alpha +at\right)\left(s+s^{\prime \prime \prime }\right)}}\\ \; & =e{\left(g_{13},g_{13}\right)}^{-\alpha (s+s^{\prime \prime \prime })}, \end{array}$$
  where $\omega =(\omega ,\tilde{\omega })$. Thus, we have the following:
  $$\begin{array}{c} NC_{b_{0}}^{\prime }\cdot \frac{\prod_{i\in T}{\left(e\left(NE_{i}^{\prime },RL\right)\cdot e\left(NF_{i}^{\prime },RK_{f(i)}\right)\right)}^{\omega_{i}^{\prime }}}{e(ND^{\prime },RK)}=NC_{b_{0}}^{\prime \prime }\cdot NC_{b_{0}}^{\prime \prime \prime }\cdot e{\left(g_{13},g_{13}\right)}^{-\alpha (s+s^{\prime \prime \prime })}=M. \end{array}$$
- Use RSK to decrypt $CRC^{\prime }$: Since the process is similar, we leave out some intermediate processes. We have the following:
  $$\begin{array}{c} \frac{\prod_{i\in T}{\left(e\left(CRE_{i}^{\prime },RL\right)\cdot e\left(CRF_{i}^{\prime },RK_{f(i)}\right)\right)}^{\omega_{i}}}{e(CRD^{\prime },RK)}=e{\left(g_{3},g_{3}\right)}^{-\alpha (s+s^{\prime \prime \prime })}. \end{array}$$
  Thus, we have the following:
  $$\begin{array}{c} CRC_{b_{0}}^{\prime }\cdot \frac{\prod_{i\in T}{\left(e\left(CRE_{i}^{\prime },RL\right)\cdot e\left(CRF_{i}^{\prime },RK_{f(i)}\right)\right)}^{\omega_{i}}}{e(CRD^{\prime },RK)}=M. \end{array}$$
- Similarly, use FSK to decrypt NC. We have the following:
  $$\begin{array}{c} \frac{\prod_{i\in T}{\left(e\left(NE_{i},FL\right)\cdot e\left(NF_{i},FK_{f(i)}\right)\right)}^{\omega_{i}}}{e(ND,FK)}=e{\left(g_{13},g_{13}\right)}^{-\alpha s}, \end{array}$$
  where $e\left(NE_{i},FL\right)=e\left(g_{13}^{a\lambda_{i}}\bar{H}{\left(f\left(i\right)\right)}^{-r_{i}},g_{123}^{\hat{t}}\right)$ and $e\left(NF_{i},FK_{f(i)}\right)=e\left(g_{13}^{r_{i}},\bar{H}{\left(f\left(i\right)\right)}^{\hat{t}}\right)$.
  Obviously, the secret key FSK can also decrypt the ciphertext NC correctly.
- Use FSK to decrypt CRC. We have the following:
  $$\begin{array}{cc} \frac{\prod_{i\in T}{\left(e\left(CRE_{i},FL\right)\cdot e\left(CRF_{i},FK_{f(i)}\right)\right)}^{\omega_{i}}}{e(CRD,FK)} & =\frac{e{\left(g_{23},g_{23}\right)}^{a\hat{t}\sum_{i\in T}\lambda_{i}\omega_{i}}}{e{\left(g_{23},g_{23}\right)}^{s(\alpha +a\hat{t})}}\\ \; & =e{\left(g_{23},g_{23}\right)}^{-\alpha s}, \end{array}$$
  where $e\left(CRE_{i},FL\right)=e\left(g_{23}^{a\lambda_{i}}\bar{H}{\left(f\left(i\right)\right)}^{-r_{i}},g_{123}^{\hat{t}}\right)$ and $e\left(CRF_{i},FK_{f(i)}\right)=e\left(g_{13}^{r_{i}},\bar{H}{\left(f\left(i\right)\right)}^{\hat{t}}\right)$.
  Then, we have the following:
  $$\begin{array}{c} CRC_{1-b_{0}}\cdot \frac{\prod_{i\in T}{\left(e\left(CRE_{i},FL\right)\cdot e\left(CRF_{i},FK_{f(i)}\right)\right)}^{\omega_{i}}}{e(CRD,FK)}=\hat{M}. \end{array}$$
  This is in the scenario of fooling the coercer: Since the fact that $CH\left(M,d_{b_{1}}\right)=CH\left(\hat{M},d_{1-b_{1}}\right)$, the coercer will believe that the message is true.
- Similarly, use FSK to decrypt $NC^{\prime }$. We have the following:
  $$\begin{array}{cc} \frac{\prod_{i\in T}{\left(e\left(NE_{i}^{\prime },FL\right)\cdot e\left(NF_{i}^{\prime },FK_{f(i)}\right)\right)}^{\omega_{i}^{\prime }}}{e(ND^{\prime },FK)}= & e{\left(g_{13},g_{13}\right)}^{-\alpha (s+s^{\prime \prime \prime })}. \end{array}$$
- Similarly, use FSK to decrypt $CRC^{\prime }$. We have the following:
  $$\begin{array}{cc} \frac{\prod_{i\in T}{\left(e\left(CRE_{i}^{\prime },FL\right)\cdot e\left(CRF_{i}^{\prime },FK_{f(i)}\right)\right)}^{\omega_{i}^{\prime }}}{e(CRD^{\prime },FK)}= & e{\left(g_{23},g_{23}\right)}^{-\alpha (s+s^{\prime \prime \prime })}. \end{array}$$
  This result confirms that our scheme supports revocability while maintaining coercion resistance.

## 6. Coercion Resistance Analysis

We will discuss in this section that our construction has coercion resistance capability. The two core tools of our scheme construction to achieve coercion resistance are composite order bilinear mapping and the chameleon hash function. The former realizes the construction of fake secret keys, and the latter realizes that the hashes of real messages and fake messages are the same.

In order to prove that our scheme satisfies coercion resistance, we need to verify that the scheme satisfies ciphertext consistency, key consistency, and the indistinguishability between real and fake messages. The detailed proof process is outlined as follows.

**Theorem** **1.** *Under the general subgroup decision assumption, our CP-ABE scheme is coercion-resistant.*

**Proof.**  To prove this theorem, we need to employ the following lemmas. □

**Lemma** **1.** *The tuple $\left\{\hat{M},1-b_{0},1-b_{1}\right\}$ and the tuple $\left\{M,b_{0},b_{1}\right\}$ are computationally indistinguishable.*

**Proof.**  The proof is straightforward because the fake message $\hat{M}$ is chosen by the IoT user before being coerced. Therefore, the user will select a fake message that is indistinguishable from the real message *M* based on the actual situation. Additionally, $b_{0}$ and $b_{1}$ are binary coins, which means that they are indistinguishable from $1-b_{0}$ and $1-b_{1}$, respectively. □

**Lemma** **2.** *The real secret key RSK and the fake secret key FSK are computationally indistinguishable.*

**Proof.**  According to the general subgroup decision assumption, we have that $g_{13}$ and $g_{123}$ are computationally indistinguishable. Then, since both *t* and $\hat{t}$ are chosen at random in $Z_{q_{123}}$, we have that α+at and $\alpha +a\hat{t}$ are computationally indistinguishable. Therefore, $RK=g_{13}^{\alpha +at}$ and $FK=g_{123}^{\alpha +a\hat{t}}$ are computationally indistinguishable, $RL=g_{13}^{t}$ and $FL=g_{123}^{\hat{t}}$ are computationally indistinguishable, and $RK_{x}=\bar{H}{\left(x\right)}^{t}$ and $FK_{x}=\bar{H}{\left(x\right)}^{\hat{t}}$ are computationally indistinguishable, which implies that RSK and FSK are computationally indistinguishable. □

**Lemma** **3.** *The normal ciphertext NC, coercion-resistant ciphertext CRC, revoked ciphertext $NC^{\prime }$, and coercion-resistant and revoked ciphertext $CRC^{\prime }$ generated in our scheme are computationally indistinguishable.*

**Proof.**  We first prove that ciphertexts NC and CRC are computationally indistinguishable. Due to the fact that *s* is chosen at random in $Z_{q_{123}}$, and due to the general subgroup decision assumption, $NC_{b_{0}}$, $NC_{1-b_{0}}$, $CRC_{b_{0}}$, and $CRC_{1-b_{0}}$ are randomly and computationally indistinguishable. Also, due to the general subgroup decision assumption, for ∀i∈[1,m], $NE_{i}$, and $CRE_{i}$, $NF_{i}$ and $CRF_{i}$ are computationally indistinguishable. Moreover, since the chameleon hash function is a general one-way hash function without knowing the trap gate, *h* and $\hat{h}$ are also computationally indistinguishable. In summary, NC and CRC are computationally indistinguishable. Ciphertext $NC^{\prime }$ is a re-randomization on ciphertext NC, so the two ciphertexts are also computationally indistinguishable. Therefore, we have that ciphertexts NC and CRC are computationally indistinguishable. Similarly, we can obtain that ciphertexts NC and $NC^{\prime }$, CRC and $CRC^{\prime }$ are also computationally indistinguishable. This completes the proof. □

According to the above three lemmas, we can easily complete the proof of the theorem.

## 7. Security Proof

We will prove in this section that our scheme satisfies semantic security. Let us first show that the revocation matrix used in this scheme is well-defined, which is identical to the revocation matrix constructed in [13]. Please refer to [13] for more details.

**Lemma** **4.** *If (A,f) and $\left(\tilde{A},\tilde{f}\right)$ are legitimate access structures for their respective LSSS schemes, we have that $\left(A^{\prime },f^{\prime }\right)$ also constitutes a legitimate access structure for an LSSS scheme.*

**Lemma** **5.** *If $\left(A^{\prime },f^{\prime }\right)$ is a legitimate access structure for an LSSS scheme. We have that both (A,f) and $\left(\tilde{A},\tilde{f}\right)$ are equally valid access structures for their corresponding LSSS schemes.*

The scheme in this paper is based on the scheme proposed by Waters [40], so we reduce the security of our scheme to the security of Waters’ scheme.

**Theorem** **2.** *Our scheme is semantically secure if Waters scheme is semantically secure.*

**Proof.**  If an adversary A can break our scheme, that means we can construct an algorithm B to break Waters’ scheme.

Algorithm B receives the public global parameter $GP_{q_{3}}$ obtained through Waters’ scheme from Challenger C:

$$\begin{array}{c} GP_{q_{3}}=\left\{g_{3},g_{3}^{a_{3}},e{\left(g_{3},g_{3}\right)}^{\alpha_{3}}\right\}, \end{array}$$

where $q_{3}$ is a prime number, and $G_{q_{3}}$, e(·,·), and $\bar{H}\left(\cdot \right)$ are utilized. For simplicity, we use different subscripts to represent different subgroups. Specifically, B does the following:

- Setup. Algorithm B picks two different prime numbers, $q_{1}$ and $q_{2}$, then constructs a group *G* of order $q_{123}$, where $G_{q_{3}}$ is a subgroup of order $q_{3}$ in *G*. Next, B requires C to generate a set of public parameters, denoted $GP_{q_{1}}$, on group $G_{q_{1}}$ using the Setup algorithm of Waters’ scheme:
  $$\begin{array}{c} GP_{q_{1}}=\left\{g_{1},g_{1}^{a_{1}},e{\left(g_{1},g_{1}\right)}^{\alpha_{1}}\right\}, \end{array}$$
  where $a_{1}$, $a_{1}\in Z_{q_{1}}$.
  Finally, B sends
  $$GP=\{g_{13},g_{1}^{a_{1}}g_{3}^{a_{3}},e{\left(g_{1},g_{1}\right)}^{\alpha_{1}}e{\left(g_{3},g_{3}\right)}^{\alpha_{3}}\},$$
  $q_{123}$, *G*, e(·,·), and $\bar{H}\left(\cdot \right)$ to A, where e(·,·) and $\bar{H}\left(\cdot \right)$ are the same as C previously sent to B. $a_{3}$ is chosen in secret and $a_{3}\in Z_{q_{3}}$, which implies that it is different from $a_{1}\in Z_{q_{1}}$. Furthermore, it holds that $g_{1}^{a_{1}}g_{3}^{a_{3}}$ can be treated as $g_{13}^{a}$, and $a\in Z_{q_{123}}$ follows the Chinese remainder theorem. Similarly, $e{\left(g_{1},g_{1}\right)}^{\alpha_{1}}e{\left(g_{3},g_{3}\right)}^{\alpha_{3}}$ can be regarded as $e{\left(g_{13},g_{13}\right)}^{\alpha }$.
- Phase1. Adversary A requests a key generation associated with attribute set *S* to B, then forwards it to the challenger C to obtain the following:
  $$\begin{array}{c} SK_{q_{3}}=\left\{S,K_{q_{3}},L_{q_{3}},{\left\{K_{x}\right\}}_{\forall x\in S}\right\}. \end{array}$$
  Next, B uses the same algorithm to generate $K_{q_{1}}$ and $L_{q_{1}}$. Finally, B sends
  $$\begin{array}{c} SK=\{S,K_{q_{1}}K_{q_{3}},L_{q_{1}}L_{q_{3}},{\left\{K_{x}\right\}}_{\forall x\in S}\}. \end{array}$$
  to A in response.
- Challege. Adversary A selects two messages $M_{0}$, $M_{1}$ and accesses structure (A,f) and sends them to B, where (A,f)∉S. B forwards them directly to C as a challenge and obtains a tuple
  $$\begin{array}{c} \left\{\left(A,f\right),M^{*}\cdot e{\left(g_{3},g_{3}\right)}^{\alpha_{3}s_{3}},D_{q_{3}},\left(E_{1,q_{3}},F_{1,q_{3}}\right),\ldots ,\left(E_{m,q_{3}},F_{m,q_{3}}\right)\right\} \end{array}$$
  from C as a reply, where $M^{*}\in \left\{M_{0},M_{1}\right\}$ is randomly chosen by C.
  Then, B picks a chameleon hash function CH and throws two binary coins, $b_{0}$ and $b_{1}$, to select two random strings, $d_{0}$ and $d_{1}$. B also needs to randomly select $\left\{r_{1},\ldots ,r_{m}\right\}\in Z_{q_{1}}$ and $s_{1}\in Z_{q_{1}}$. Then, B obtains a set of values $\left\{\lambda_{1}^{\prime },\ldots ,\lambda_{m}^{\prime }\right\}$ through calculation. Finally, B outputs and sends ciphertext CT to A in the following form:
  $$\begin{array}{c} CT=\{\left(A,f\right),C_{0},C_{1},D,\left(E_{1},F_{1}\right),\ldots ,\left(E_{m},F_{m}\right),CH,d_{0},d_{1},h\}, \end{array}$$
  with the following components defined as:
  $$\begin{array}{cc} \; & C_{b_{0}}=M^{*}\;\cdot \;e{\left(g_{1},g_{1}\right)}^{\alpha_{1}s_{1}}e{\left(g_{3},g_{3}\right)}^{\alpha_{3}s_{3}},C_{1-b_{0}}\overset{R}{\leftarrow }G_{T},D=D_{q_{3}}\;\cdot \;{\left(g_{1}\right)}^{s_{1}},\\ \; & E_{i}=E_{i,p_{3}}\;\cdot \;{\left(g_{1}\right)}^{a_{1}\lambda_{i}^{\prime }},F_{i}=F_{i,p_{3}}\;\cdot \;{\left(g_{1}\right)}^{r_{i}^{\prime }},\forall i\in \left[1,m\right],\\ \; & h=CH\left(M_{0},d_{b_{1}}\right)=CH\left(M_{1},d_{1-b_{1}}\right). \end{array}$$
  Similarly, according to the Chinese remainder theorem, A will treat the secret values $s_{1}\in Z_{q_{1}}$ and $s_{3}\in Z_{q_{3}}$ in ciphertext CT as $s\in Z_{q_{123}}$. More importantly, since A does not know the trapdoor of the chameleon hash function CH, A considers the chameleon hash function to be a general one-way hash function.
- Phase2. A continues to send key generation queries to B, and B responds as in Phase 1.
- Guess. Finally, A sends $b^{\prime }$ to B as a guess, and B forwards the result directly to C.

If A can gain advantages in the above games, B can leverage the advantages gained by A to break Waters scheme. □

## 8. Performance and Evaluation

In this section, we conduct a detailed performance evaluation of our proposed scheme. The implementation utilizes the Java Pairing-Based Cryptography (JPBC) library [41], supplemented by a C library-based wrapper for the Pairing-Based Cryptography (PBC) library [42], which enhances cryptographic operations. All experiments were carried out on a desktop machine configured with an Intel(R) Core(TM) i5-10400F CPU operating at 2.90 GHz, featuring a shared 12 MB L3 cache and 16 GB of DDR4 memory clocked at 2400 MHz.

We primarily simulated the execution times of the algorithms in the data transmission phase, data reception phase, and policy revocation phase of our scheme, comparing them with the corresponding algorithms proposed in [13,17]. The evaluation criteria are based on computational efficiency, as these three phases are particularly sensitive to time. Since the algorithms EncAlgo1 and EncAlgo2 in our scheme exhibit the same efficiency in the data transmission phase, we only simulated the algorithm EncAlgo2. Additionally, we simulated the algorithms DecAlgo1 and RevokeAlgo.

Our experimental results show that the encryption and decryption algorithms in our scheme perform similarly to those in [17], while demonstrating an approximately 50% performance improvement over the scheme proposed in [13], showcasing competitive efficiency. This result is expected, as the ciphertext size in our scheme is half that of [13] while being nearly identical to that in [17]. Additionally, the performance of all three schemes increases with the number of attributes involved in the operations, which allows our scheme to exhibit a more significant advantage in scenarios involving large numbers of attributes. In the revocation process (Algorithm RevokeAlgo), we only compared our scheme with the algorithm from [13], as the scheme in [17] does not support revocation. Experimental results show that our scheme outperforms the revocation algorithm in [13], with a speed improvement of approximately two-fold. This enhancement is mainly due to the fact that our ciphertext size is half that of [13]. This performance improvement is especially critical in environments where revocation is frequently required, as it directly affects the system’s overall responsiveness and scalability.

**Figure 2:**
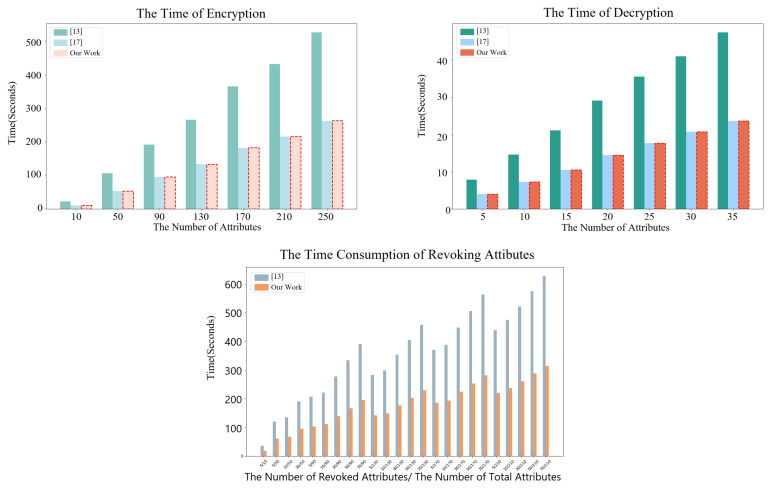
Comparison of experimental simulations [13,17].

The detailed experimental results are shown in Figure 2, which presents a performance comparison across different attribute counts and operations. It is important to note that we did not include efficiency measurements for the system initialization phase and user key generation phase in this experiment, as these processes typically occur during the initialization stage and are not considered time-critical operations. Therefore, they have a negligible impact on the overall system performance during regular operation.

The experimental results are derived based on the aforementioned desktop computing environment, indicating that our scheme is suitable for IoT devices with relatively high computational capabilities, such as smart gateways, smart routers, smartphones, and sensors with integrated computational power. However, the scheme involves two types of computationally intensive operations, namely bilinear mapping and modular exponentiation in groups, which impose certain limitations on its direct applicability to low-power IoT devices, such as simple sensor nodes or low-performance embedded devices. Fortunately, this limitation can be effectively mitigated by incorporating hardware acceleration (e.g., cryptographic accelerators, FPGAs, or ASICs) or adopting distributed computing strategies, enabling the scheme to support resource-constrained devices. Additionally, by leveraging computation outsourcing, complex computational tasks can be offloaded to cloud services or other high-performance computing modules, facilitating the deployment of the scheme on low-power IoT devices [43]. Therefore, the proposed scheme is not only well-suited for high-performance IoT devices but can also be extended to resource-constrained scenarios through the aforementioned optimization strategies.

In summary, the experimental results validate the superior performance and practicality of our proposed scheme, particularly in environments with dynamic attribute changes and frequent revocation requirements. The optimized handling of encryption, decryption, and revocation processes makes our scheme highly suitable for real-world applications that demand both security and efficiency.

## 9. Summary and Outlook

This paper proposes a new CP-ABE scheme designed to enhance data security and privacy in the IoT. The proposed scheme addresses the shortcomings of existing ABE systems in defending against coercion attacks. In addition to strengthening coercion resistance, the scheme introduces a revocation mechanism to dynamically manage and revoke user access rights based on changes in IoT user attributes. This mechanism significantly enhances the flexibility and security of access control, particularly in environments with dynamic user roles and permissions, demonstrating superior adaptability and scalability.

However, despite the robust coercion resistance and effective access control management demonstrated by the proposed scheme, there are several potential limitations and shortcomings. Firstly, as the number of users and attributes involved in the system increases, the encryption and decryption processes will incur higher computational and storage overhead. This may affect system performance, particularly in large-scale IoT environments. Therefore, future research could focus on optimizing the efficiency of the algorithms and reducing computational and storage requirements to improve the scheme’s usability in large-scale settings.

Secondly, although the attribute revocation mechanism can dynamically manage user access rights, frequent revocation operations may increase system complexity and response time. Thus, designing efficient revocation algorithms and reducing latency in scenarios with high-frequency revocations remains a critical issue for future research.

Lastly, while the proposed scheme offers resistance against coercion attacks, its security relies heavily on secure key management and storage mechanisms. If key leakage or attacks occur, the security of the entire system could be compromised. Therefore, future work could explore more robust key management solutions, such as integrating Trusted Execution Environments (TEE) or blockchain technology, to further enhance system security.

In summary, the proposed scheme demonstrates theoretical feasibility and practical value in enhancing data security, protecting user privacy, and enabling flexible access control in IoT environments. However, optimizing its performance in large-scale IoT settings and improving its adaptability in highly dynamic environments will be key areas for future research.

## Data Availability

The original contributions presented in this study are included in the article Further inquiries can be directed to the corresponding author.
